# Experimental Comparison of the Performance of Cutting Bone and Soft Tissue between Piezosurgery and Conventional Rotary Instruments

**DOI:** 10.1038/s41598-018-35295-6

**Published:** 2018-11-21

**Authors:** Yoshio Otake, Megumi Nakamura, Akiko Henmi, Tetsu Takahashi, Yasuyuki Sasano

**Affiliations:** 1 Division of Oral and Maxillofacial Surgery, Tohoku University Graduate School of Dentistry, Sendai, Japan; 2 Division of Craniofacial Development and Regeneration, Tohoku University Graduate School of Dentistry, Sendai, Japan

## Abstract

Piezosurgery is an innovative technique widely used for osteotomies in the field of oral and maxillofacial surgery. The surgical technique has been clinically supposed to cut mineralized bone selectively with reducing the risk of damage to adjacent soft tissues. However, none of the previous literature has reported any evidence of scientific experiments to examine performance of the piezoelectric device, *i.e*. the time required for cutting bone and the effect on soft tissues under the standardized conditions. This study was designed to test the hypothesis that cutting time of the piezoelectric device is longer than that of rotary instruments while the cut surface of bone is smoother and soft tissues are less damaged with piezosurgery under the standardized experimental system. We measured the time for cutting bone and soft tissues of rats with the piezoelectric device and rotary instruments. Damage to soft tissues was examined histologically, and the cut surface of bone was investigated using scanning electron microscopy. Our study demonstrated experimentally that piezosurgery provides a smooth cut bony surface with no damage to soft tissues and takes longer time to cut bone than conventional drillings. We propose that piezosurgery is beneficial for medical safety and usability.

## Introduction

Piezosurgery is an innovative osteotomy technique using piezoelectric ultrasonic vibrations. Ultrasonic vibrating instruments for cutting mineralized tissue have been reported since the 1950s^1,2^. In 1988, Italian oral sugeon, Tomaso Vercellotti, developed a piezoelectric osteotomy device, which provided the opportunity for the widespread clinical use of piezosurgery^3,4^. Currently, piezoelectric devices are used widely for osteotomies, such as maxillary sinus lift, impacted mandibular third molar extraction, and bone grafting, in the field of oral and maxillofacial surgery^2,4^.

The major advantage of piezosurgery is its selective cutting of mineralized bone. Frequencies higher than 50 kHz are needed to cut soft tissues. The piezoelectric device is designed to produce ultrasonic microvibrations of 60–210 μm at a frequency of 25–30 kHz. Thus, piezoelectric devices, different from conventional rotary instruments and microsaws, are able to cut only mineralized tissues^5–7^. Therefore, piezosurgery can reduce considerably the risk of damage to adjacent soft tissues, such as blood vessels, nerves, and mucous membranes, in cases of osteotomy^8–11^. Other advantages of piezosurgery include reduction of overheating resulting from the generation of a cavitation effect and better visibility of the surgical area due to less bleeding^2,12,13^. Moreover, use of piezosurgery for extraction of the impacted mandibular third molar was reported to produce less facial swelling and trismus postoperativily compared to that of rotary osteotomy^14,15^. Recent studies on the healing of bone defects experimentally created by piezosurgery demonstrated no difference in the newly formed bone volume and the healing process between piezosurgery and conventional osteotomy techniques^16,17^. Meanwhile, piezosurgery was shown to have a longer surgical time than conventional osteotomies^15,18^.

Most studies on comparison of piezosurgery and conventional osteotomies have focused on postoperative outcomes, and little is known about the cutting performance of the piezoelectric device itself. With respect to the time required for cutting, surgical time in clinical cases has been investigated to date, while little information is available about experimentally measured cutting time with the piezoelectric device compared to conventional osteotomy instruments. Furthermore, few studies also have reported the effect of piezosurgery on soft tissues.

The present study was designed to test the hypothesis that cutting time of the piezoelectric device is longer than that of rotary instruments while the cut surface of bone is smoother and soft tissues are less damaged with piezosurgery under the condition of the standardized cutting force, 1.5 N, which was recommended in a previous study^19^. We characterized piezosurgery by comparing it to conventional drillings. We established an experimental system to assess cutting performance using a force gauge and measured the time required for cutting. We also examined the damage to soft tissues with histology and the cutting surface morphology of bone with scanning electron microscopy (SEM).

## Results

### Fluctuation of cutting force

**Figure 1:**
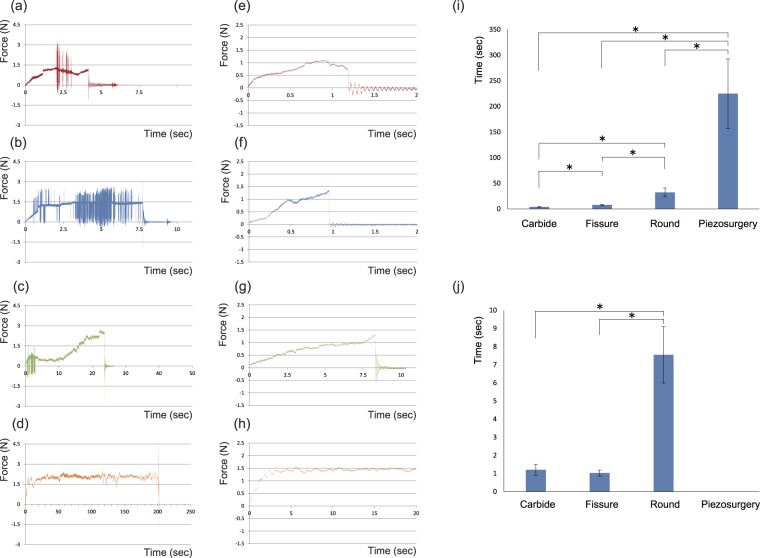
Measurement of the time required for cutting the tibia and tongue. (**a**–**d**) Force-time graph of tibias: (**a**) Carbide bur, (**b**) Fissure bur, (**c**) Round bur, and (**d**) Piezosurgery. (**e**–**h**) Force-time graph of tongues: (**e**) Carbide bur, (**f**) Fissure bur, (**g**) Round bur, and (**h**) Piezosurgery. (**i**) Comparison of time required to cut the tibia among the instruments (*n* = 6; Games-Howell test, *P* < 0.05). Carbide and fissure burs (*P* = 0.001), carbide and round burs (*P* = 0.002), fissure and round burs (*P* = 0.003), fissure bur and piezosurgery (*P* = 0.002), round bur and piezosurgery (*P* = 0.003). (**j**) Comparison of time required to cut the tongue among the instruments (*n* = 6; Games-Howell test, *P* < 0.05). Carbide and fissure burs (*P* = 0.454), carbide and round burs (*P* < 0.001), fissure and round burs (*P* < 0.001). The result for piezosurgery was not shown because the tongue was not cut within 20 s.

Samples from six rats were cut with the piezoelectric device and conventional rotary instruments; that is, carbide, fissure, and round burs. The time for cutting is shown in the graph for typical samples (Fig. 1a–h).

The force was stable and fluctuation was limited to approximately 1.5 N until the tibia was cut through with the piezoelectric device (Fig. 1d), while the force fluctuated widely with the rotary instruments (Fig. 1a–c). Conventional drillings took a few seconds to cut through the tongue with a force of less than 1.5 N (Fig. 1e–g). In contrast, piezosurgery showed limited fluctuation of pressure for the tongue as for the tibia, but did not cut the tongue (Fig. 1h).

### Time required for cutting

The time required to cut through the tibia through is shown in Table 1. Piezosurgery took the longest (average 255.06 s), while the carbide bur took the shortest (average 3.94 s) time, and the round bur took the longest time among the conventional drillings (average 32.44 s).

**Table 1:** Time required for cutting the tibia and tongue (n = 6).

		Carbide	Fissure	Round	Piezosurgery
Tibia	Time(sec)	4.44	7.86	30.15	204.01
		3.02	6.15	23.76	202.06
		4.56	6.87	45.1	345.8
		3.42	7.76	39.74	139.65
		3.99	9.71	24.28	238.57
		4.18	8.99	31.62	220.29
	Mean	3.94	7.89	32.44	225.06
	SD	0.6	1.31	8.5	67.9
Tongue	Time(sec)	1.53	1.34	7.37	*
		1.19	1.04	8.34	*
		1.45	0.96	9.98	*
		1.38	0.85	5.77	*
		0.93	1.01	6.04	*
		0.77	0.98	7.81	*
	Mean	1.21	1.03	7.55	—
	SD	0.3	0.17	1.55	—

*Time for piezosurgery was not shown because the tongue was not cut within 20 seconds.

The time required to cut through the tongue is also shown in Table 1. The fissure bur took the shortest (average 1.03 s) and the round bur took the longest (average 7.55 s) time. Piezosurgery did not cut the tongue.

The time required for cutting was analysed statistically and is shown in Fig. 1i,j. The time for piezosurgery to cut through the tibia was the longest and was significantly different from that for the carbide (*P* = 0.002), fissure (*P* = 0.002), and round (*P* = 0.002) burs. The time for the carbide bur was the shortest among the conventional drillings and was significantly different from that for the fissure (*P* = 0.001) and round (*P* = 0.002) burs. The round bur took longer than the fissure bur (*P* = 0.002).

The times for the carbide and fissure burs were not statistically different. The round bur took longer than the carbide (*P* < 0.001) and the fissure (*P* < 0.001) burs. Piezosurgery did not cut the tongue.

### The region of tongue to which each instrument was applied

**Figure 2:**
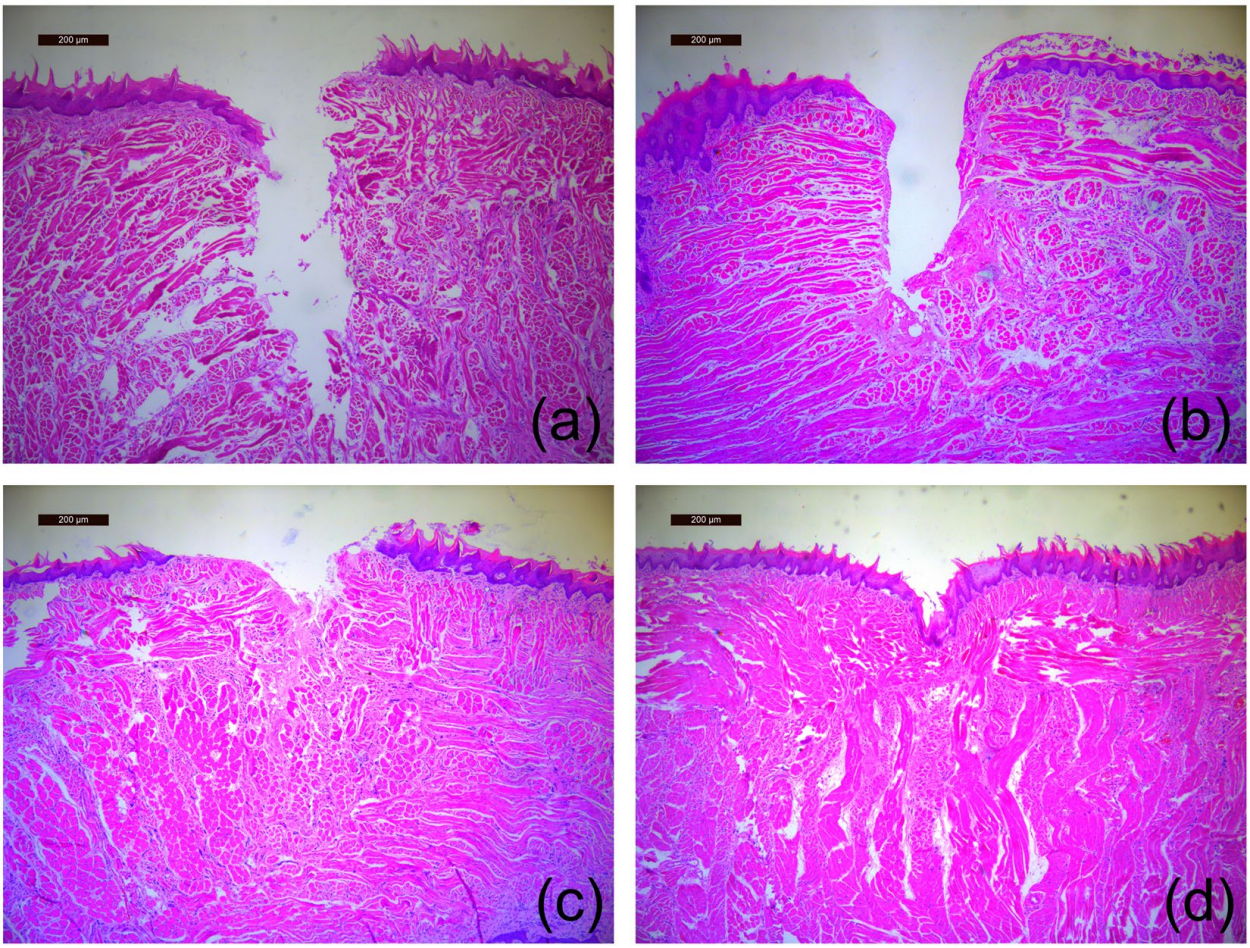
Histology of the region of the tongue to which each instrument was applied (H-E staining, scale bar = 200 μm). (**a**) Carbide bur, (**b**) Fissure bur, (**c**) Round bur, and (**d**) Piezosurgery. The damage caused by the conventional drillings extends from mucous epithelia through submucous and muscular layers. Piezosurgery made a dent but did not damage any tissue.

The region of the tongue to which each instrument was applied was examined histologically. Damage caused by the rotary instruments extended from mucous epithelia through submucous and muscular layers (Fig. 2a–c). The carbide and round burs showed large irregular (Fig. 2a) and shallow and irregular (Fig. 2c) damaged regions, respectively, and the fissure bur caused a relatively smooth surface (Fig. 2b). Piezosurgery made a dent, but did not damage any tongue epithelia or lingual papilla (Fig. 2d).

### Bony regions to which each instrument was applied

**Figure 3:**
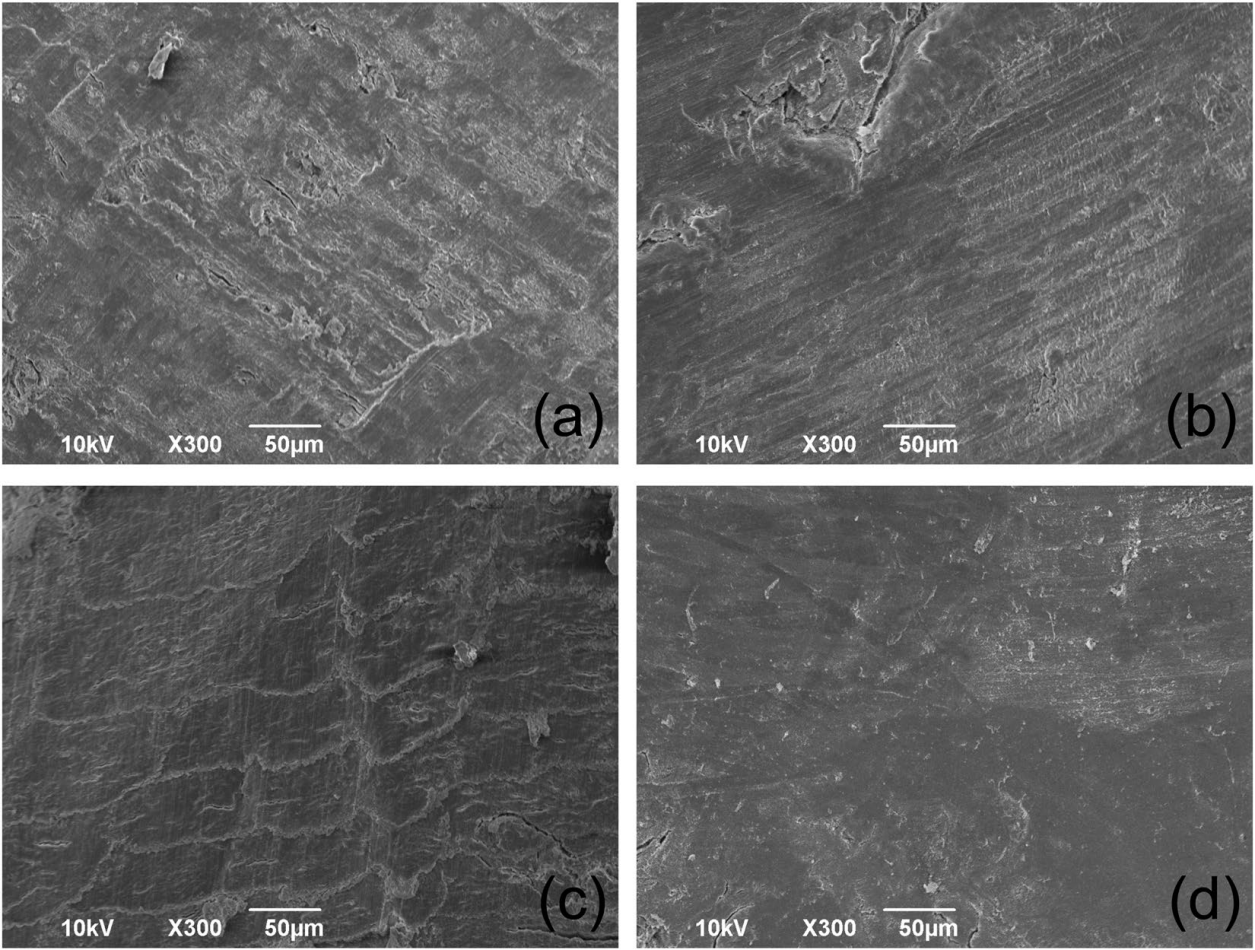
SEM images of the tibia to which each instrument was applied. (**a**) Carbide bur, (**b**) Fissure bur, (**c**) Round bur, and (**d**) Piezosurgery. The smooth surface without damage was made by piezosurgery, while conventional drillings caused the rough surfaces.

SEM showed a smooth surface due to the piezoelectric device (Fig. 3d). The carbide bur caused a rough surface with bone debris (Fig. 3a). The fissure bur made fine line scratches (Fig. 2b). The round bur caused a scale-like surface (Fig. 2c).

## Discussion

It has been reported clinically that piezosurgery cuts only bone but does not damage soft tissues^5–7^. Piezosurgery has been proposed as a minimally invasive surgical technique in cranial base and spine surgery^20^. To our knowledge, our study confirmed the reported clinical advantage of piezosurgery experimentally, *i.e*. giving no damage to soft tissues for the first time. Conventional drillings cut through the tongue within 10 s, while piezosurgery only made a dent on the tongue surface and cut none of the examined tongue samples (Table 1; Figs 1j and 2d). The tongue was not cut even though the time was extended by 200 s (data not shown). Piezosurgery is beneficial for protection of soft tissues during bone surgery.

Various clinical trials in the published literature have been reviewed systematically to evaluate the clinical efficacy of peizosurgery with meta-analyses. A systematic review indicated that piezosurgery can reduce the risk of the Schneiderian membrane perforation in maxillary sinus floor augmentation compared to conventional rotary osteotomy^21^. Another review examined outcomes for the surgical extraction of mandibular third molars and found a lower risk of neurological complications with the piezoelectric device than with conventional rotary burs^22^. A recent report showed less postoperative neurosensory disturbance after orthognathic surgery with piezosurgery compared to conventional techniques^23^. These meta-analyses suggested that piezosurgery does not damage soft tissues, which has been supported by our results of the present study.

The operative field of osteotomies and bone biopsies in piezosurgery is said to have bleeding from surrounding soft tissues reduced significantly and the operative field almost free of blood during the cutting compared to conventional drillings, where blood is moved in and out of the cutting area and visibility is decreased^4^. It has been supposed to be due to the cavitation effect created by the cooling fluid distribution and/or by the type of vibration the instrument generates, in which the blood is essentially washed away. Our results of the experiments demonstrated that piezosurgery does not damage soft tissues and provided the scientific supports for the clinical empirical concepts for the first time.

Previous reports suggested that piezosurgery took longer to cut bone compared to conventional drillings, most of which described the time required for clinical surgery but did not compare the performance of the instruments experimentally^15,18^. Therefore, our study established the original experimental model to measure the time required for cutting standardized samples (e.g., tibia and tongue of 10-week-old rats) and compared results among the instruments. Our results indicated that conventional drilling cut through the tibia within one minute, while piezosurgery took longer than three minutes (Table 1; Fig. 1i). Our study also demonstrated experimentally that piezosurgery took longer to cut bone compared to conventional drillings.

The force to cut the tibia fluctuated widely with conventional drillings, although we attempted to keep a constant pressure of approximately 1.5 N (Fig. 1a–c). This may be due to fluctuation made by the burs while they kept contact with the samples. The round bur might have fluctuated widely at the beginning of cutting and the fluctuation decreased later when the bur fit the defect it made. In contrast, fluctuation of piezosurgery was limited compared to the conventional drillings (Fig. 1d), which suggests that piezosurgery is able to cut bone with a stable force with little fluctuation of the insert tip of the piezoelectric device.

The present study examined the bony regions to which each instrument was applied using SEM. As a result, piezosurgery caused a smooth surface without bone debris (Fig. 3) compared to other conventional drillings, which supports a report of equivalent rabbit experiments made by Maurer and colleagues^24^. It has not been known how piezosurgery affects viability and function of cellular components. The viability and differentiation potency of derived osteogenic cells showed no difference between autogenous bone chips harvested with piezosurgery and those with conventional drillings^25^. Osteocytes were maintained in the cut region made by piezosurgery and conventional drillings^26^. Previous studies suggested that there are no significant differences in healing of osteotomy sites between piezosurgery and conventional drillings^16,17^. The smear layer left on the bone surface by piezosurgery may even impair bone healing^27^. Preparation of the smooth cut surface may not contribute to maintaining cell viability and differentiation potency, and further investigations are required to provide better understanding.

There are some limitations to this study. Firstly, the cutting force in the experiments was 1.5 N and the force larger than 1.5 N was not applied. The larger cutting force may damage the tongue mucosa and reduce the cutting time. Secondly, we used only the OT-7 tip for piezosurgery. The use of different tips may cause different results of damage to the mucosa and cutting time. Other conditions of cutting force and tips should be examined for cutting time and damage given to the mucosa in the future.

Our study demonstrated experimentally cutting time of the piezoelectric device is statically longer than that of conventional rotary instruments while piezosurgery provides a smooth cut bony surface with no damage to soft tissues under the condition of the standardized cutting force, 1.5 N. Based on these results, we propose that piezosurgery is beneficial for medical safety and usability.

## Methods

### Animals and sample preparation

All experimental procedures conformed to “Regulations for Animal Experiments and Related Activities at Tohoku University”, and were reviewed by the Institutional Laboratory Animal Care and Use Committee of Tohoku University, and finally approved by the President of the University.

We obtained 10-week-old male Wistar rats weighing 210–230 g from the SLC Corporation (Japan SLC, Inc., Hamamatsu, Japan). They were euthanized with an overdose of isoflurane by inhalation. Tibias and tongues were resected as samples.

### Osteotomy instruments

For piezoelectric osteotomies, Piezosurgery Touch® with an OT-7 tip (Mectron, Carasco, Italy) was used with a frequency of 30 kHz (special mode) under 20 mL/min irrigation. For conventional drilling, VOLVERE Vmax® (Nakanishi, Inc., Kanuma, Japan) with carbide, fissure and round burs was used at 20 × 1000 rpm under irrigation.

### Measurement of cutting time

A force gauge (ZTS-50N; Imada Co., Ltd., Toyohashi, Japan) and a force-time graphing software (Force Recorder Standard; Imada Co., Ltd., Toyohashi, Japan) were used to measure the time required for cutting samples. A force gauge was equipped with a handpiece holder set at a distance of 5 cm from an axis of the force gauge to the tip of the insert tip or burs (Fig. 1). Using each instrument, tibias were cut transversely proximally at a site 10 mm away from the junction of the tibia with the fibula. Tongues were divided into two halves at the midline, and halves were cut transversely at a site 10 mm from the apex. The time required for cutting through tibias and tongues at approximately 1.5 N with the piezoelectric device or rotary instruments was measured.

### Statistical analysis

Statistical analysis was performed with SPSS 22.0 (IBM Corp., Armonk, NY, USA) to compare the time required for cutting samples between the piezoelectric device and rotary instruments. Data were compared by one-way analysis of variance (ANOVA) followed by the Games-Howell test. The significance level was set at 0.05.

### Histology

The tongue samples were fixed in 4% paraformaldehyde in 0.1 M phosphate buffer, pH 7.4. After dehydration through a graded series of ethanol solutions, samples were embedded in paraffin. Sections 5μm thick were cut and processed for hematoxylin-eosin (H-E) staining.

### Surface morphology by SEM

Proximal and distal epiphyses of tibias were cut and bone marrow was removed with a wire 0.3 mm in diameter. After irrigation of the bone marrow cavity with physiological saline, tibias were cut transversely proximally at a site 10 mm away from the junction of the tibia with the fibula by piezosurgery or conventional drilling. Eventually, 3–4 mm long specimens were prepared for SEM. Specimens were fixed in 2% paraformaldehyde with 2% glutaraldehyde in 0.1 M phosphate buffer, pH 7.4, dehydrated, replaced with isoamyl acetate, and dried using a critical point dryer (JCPD-5; JEOL, Tokyo, Japan). The dried specimens were coated with platinum and the surface morphology was examined by scanning electron microscope (SEM) (JSM-6390LA, JEOL, Tokyo, Japan).

## Data Availability

All data generated or analysed during this study are included in this published article.
